# Stakeholder insights into implementing a systems-based suicide prevention program in regional and rural tasmanian communities

**DOI:** 10.1186/s12889-022-14721-5

**Published:** 2022-12-12

**Authors:** Laura Grattidge, Terry Purton, Stuart Auckland, David Lees, Jonathan Mond

**Affiliations:** 1 Centre for Rural Health, University of Tasmania, Locked Bag 1322, TAS 7250 Launceston, Australia; 2 School of Nursing, University of Tasmania, Launceston, Australia; 3 School of Medicine, Western Sydney University, Sydney, Australia

**Keywords:** Suicide prevention, Systems-based approach, Regional and rural, Community, Evaluation

## Abstract

**Purpose:**

With emerging evidence indicating that systems-based approaches help optimise suicide prevention efforts, the National Suicide Prevention Trial sought to gather evidence on the appropriateness of these approaches to prevent suicide among at-risk populations, in regional and rural communities throughout Australia. The Tasmanian component of the Trial implemented the LifeSpan systems framework across three distinct rural areas with priority populations of men aged 40–64 and people 65 and over. The University of Tasmania’s Centre for Rural Health undertook a local-level evaluation of the Trial.

**Aims:**

To explore key stakeholder perceptions of implementing a systems-based suicide prevention program in regional and rural communities in Tasmania, Australia.

**Method:**

This study utilised qualitative methods to explore in depth, stakeholder perspectives. Focus groups and interviews were conducted with 46 participants, comprising Trial Site Working Group members (*n* = 25), Tasmania’s Primary Health Network employees (*n* = 7), and other key stakeholders (*n* = 14). Approximately half of participants had a lived experience of suicide. Data were thematically analysed using NVivo.

**Results:**

Key themes centred on factors impacting implementation of the Trial. These included how the Trial was established in Tasmania; Working Group governance structures and processes; communication and engagement processes; reaching priority population groups; the LifeSpan model and activity development; and the effectiveness, reach and sustainability of activities.

**Discussion:**

Communities were acutely aware of the need to address suicide in their communities, with the Trial providing resources and coordination needed for community engagement and action. Strict adherence to the Lifespan model was challenging at the community level, with planning and time needed to focus on strategies influencing whole or multiple systems, for example health system changes, means restriction. Perceived limitations around implementation concerned varied community buy-in and stakeholder engagement and involvement, with lack of role clarity cited as a barrier to implementation within Working Groups. Barriers delivering activities to priority population groups centred around socio-cultural and technological factors, literacy, and levels of public awareness. Working Groups preferred activities which build on available capital and resources and which meet the perceived needs within the whole community. Approaches sought to increase awareness of suicide and its prevention, relationships and partnerships, and the lived experience capacity in Working Groups and communities.

**Conclusion:**

Stakeholder insights of implementing the National Suicide Prevention Trial in regional and rural Tasmanian from this study can help guide future community-based suicide prevention efforts, in similar geographic areas and with high-risk groups.

**Supplementary Information:**

The online version contains supplementary material available at 10.1186/s12889-022-14721-5.

## Introduction

Suicide is a major public health problem, with [1] rates in Australia steadily increasing over the last decade [1, 2]. There are personal costs of suicide, with those impacted by suicide profoundly and negatively affected, the social costs and ripple effects into whole communities, and financial costs to individuals and the Australian economy, with a conservative estimate of the cost of suicide and suicidal behaviour as $17.5 billion [3]. Impacts of suicide throughout Australian communities extend across all cultures, societies and systems [2, 4, 5]. Approaches seeking to address the factors contributing to suicide across all levels of society, are based on the premise that suicide and its prevention are situated within complex, multilevel systems, requiring a range of strategies to be simultaneously implemented [6–8]. Trials of systems approaches to suicide prevention are in their infancy [7–10], with recent trials [11–13] including the European Alliance Against Depression in Europe and Zero Suicide in the United States, both supporting preliminary findings of reduced suicide and suicidal behaviours [8, 11–16]. Research [17] suggests multifactorial nature of systems approaches means they are also more complex to implement, and evidence as to their utility in areas of regional, rural and remote areas of the country remains limited [17, 18].

In 2016, the Australian Government initiated the National Suicide Prevention Trial (the Trial), recognising a need for building the evidence base on suicide prevention in regional Australia. Specifically, exploring how systems-based approaches can be used to address the complex issue of suicide, at the local community-level, with population groups most at risk [15, 16]. Twelve sites across Australia were selected for the Trial, with Primary Health Networks receiving funding over four years to coordinate efforts within their local communities. Sites were selected by the Australian Government, based on community-level factors, including local suicide rates, existing local resources, and capacity to implement initiatives [15, 19].

Tasmania was selected as one of the trial sites [19]. Australia’s only island state and home to approximately 540,000 people, in 2020 Tasmania had the second highest rate of suicide in Australia (15.9 per 100,000), second to the Northern Territory (20.4) [20]. Between 2012 and 2018, rates of suicide in Tasmania were highest for people aged 35 to 44 years, with rates for men four times higher than for women, similar to other areas of Australia [21]. Causes attributing to suicide rates in Tasmania included people experiencing contextual or situational stressors (including experience of abuse or violence and substance abuse or misuse), mental and/or physical ill health, contact with the legal system and access to social and health services [22, 23].

When exploring rurality, the Modified Monash (MM) Model remoteness classification can be used to measure remoteness, on a scale from category MM1 (major city) to MM7 (very remote). The Tasmanian capital city of Hobart is classified as MM2 (regional centre), as is the second largest city of Launceston. The remaining parts of the state range between MM2 to MM5 (regional centres to small rural towns). Making up the Tasmanian trial site were the Local Government Areas (LGA) of Launceston in Northern Tasmania (regional centre with around 66,000 people), Break O’Day on the North-East coast (comprising scattered small towns of around 1,000 to 5,000 people, and large rural towns, between 15,000 and 50,000 people), and the North-West Coast LGAs of Devonport, the Central Coast and Burnie (large rural towns) [24]. Site selection was made by the sole Tasmanian Primary Health Network and was based on consideration of existing physical and social infrastructure and capital, resources and suicide prevention activities, community suicide risk factor profiles, and local capacity to be involved [25]. Host organisations included a local council (Launceston), a local neighbourhood house network (Break O’Day), and a not for profit organisation (North-west) [25, 26]. Working Groups were convened for each of the trial site locations to plan and undertake activities and were comprised of a funded coordinator, chair, host organisation manager, local community members including people with lived experience of suicide, and representatives from service organisations and Tasmania’s Primary Health Network [27, 28].

The priority population groups for Tasmania were men aged 40 to 64 years and people 65 years and over. These groups were chosen by the Tasmanian Suicide Prevention Trial Advisory Group (TSPTAG)—the program’s overarching governing committee convened by the Primary Health Network, representing local service providers, peak bodies, and stakeholders. Priority populations were chosen based on a number of factors including analysis of local data on suicide deaths, attempts, self-harm and related risk factors and sufficient numbers of people and available services for these priority groups in each of the regions [25, 26, 29].

**Fig. 1:**
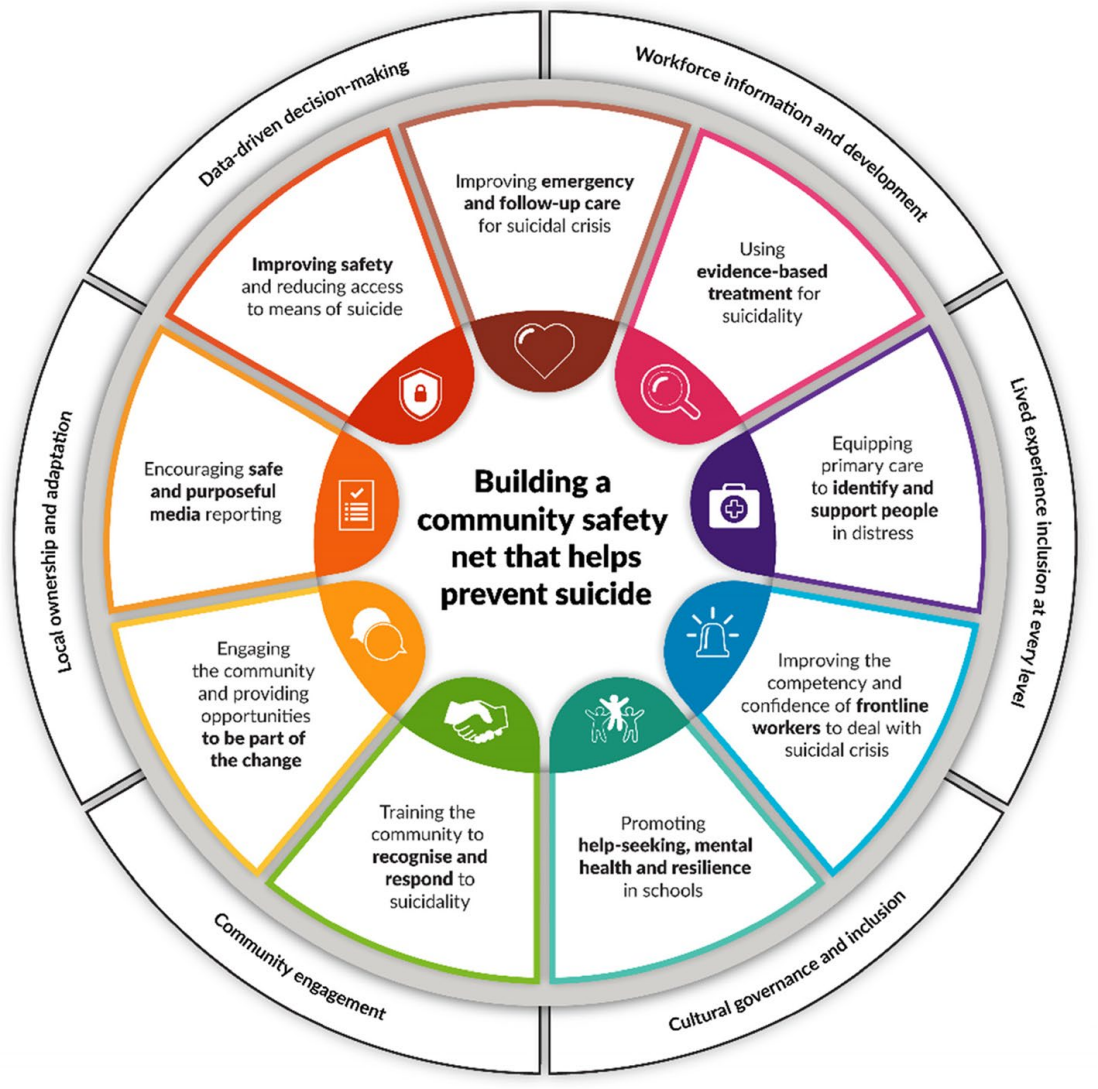
National Suicide Prevention Trial in Tasmania – The LifeSpan Framework. Source. LifeSpan model(16) from Black Dog Institute

The system’s-based model, also selected by the TSPTAG, was the Black Dog Institute’s *LifeSpan* framework [7, 25], which combines nine strategies, for which available evidence was deemed strongest at the time of framework development. LifeSpan is grounded in six ‘building blocks’ or guiding principles, which, together with the nine strategies, ultimately aim to build whole of community capacity to better support people facing suicidal crisis [8, 30, 31]. Research shows reduced suicide rates and attempts as a result of the simultaneous implementation of this framework, incorporating businesses, health, frontline services, education, and the community [7, 29, 32] (Fig. 1).

The literature evaluating suicide prevention initiatives in Australia has largely focused on the utility of specific training or intervention programs or isolated strategies, rather than the feasibility of broad or synergistic (systems-based) approaches. Further, evidence evaluating the effectiveness of community-led programs in regional, rural and remote regions of Australia is particularly limited [27, 33–36]. To help build this evidence base, the University of Tasmania’s Centre for Rural Health were engaged to evaluate the Tasmanian trial site, as a process-oriented evaluation supplementing a more outcome-oriented, national-level evaluation conducted by the University of Melbourne. A Participatory Action Research (PAR) approach was employed as a reflective process of collaborating with Working Groups in the design and conduct of evaluation activities (reported as preliminary findings of the Trial in Tasmania (excluding interview and focus group data) and a separate commentary from the perspectives of the evaluation team [25, 26]. This approach aligns with the literature highlighting the importance of taking into account both a collaborative approach and public opinion when developing, refining, implementing, and evaluating health promotion programs [28, 30]. A PAR approach has the potential to promote ownership of programs, transfer power from the researchers to the researched, and enable sustainability of efforts [31]. The evaluation aimed to build the evidence base by exploring the views of key stakeholders involved with the Trial in Tasmania, particularly in relation to the implementation of suicide prevention activities under the LifeSpan systems-based approach. Findings may help inform future community based suicide prevention interventions in regional and rural areas, focused on at-risk population groups, and using a systems-based approach.

## Methodology

### Design

This study utilised a qualitative research design, with [37] interviews and focus groups complementing quantitative and observational data collected and reported in previous studies [25, 26]. Interviews and focus groups allow for deeper inquiry into participants’ experiences with the Trial, allowing theoretically relevant information to emerge from the data. Similar qualitative approaches are commonly used in suicidology to give voice to participants, and in particular stakeholders, community members and people with lived experience of suicide [38, 39]. As previously mentioned, a PAR approach was utilised throughout all stages of the evaluation, as a reciprocal process of action and reflection, to guide the design and conduct of evaluation activities [25, 26].

### Participants

Participants comprised 46 people involved with the Trial across the three sites., including Working Group members (*n* = 25), project staff at Tasmania’s Primary Health Network (*n* = 7), and several external stakeholders, including representatives of the national evaluation team, peak suicide prevention body representatives, TSPTAG members, and members of the Tasmanian Suicide Prevention Community Network (*n* = 14). Participants were invited by the research team to participate in the study (either an interview or focus group) through email, and also asked to share the study invitation with others whom they felt had similar experiences with the Trial. Participants were selected based on the aims of the study and their ability to provide unique and rich information of value to the study, in this case, based on their experiences or role with the Trial [40]. Participant ages ranged from 28 to 75 years (median = 53 years, *SD* = 11.7). Just over half identified as female (51.1%) and the majority (53.3%) reported a lived experience of suicide, as defined by Roses in the Ocean [41].

### Data collection and analysis

Data were collected between January and March 2020 across the three trial site locations. Participants were provided with a participant information sheet and were required to provide written informed consent prior to participation. Focus groups and interviews were guided by semi-structured questions relating to key governance processes and structures, including decision making, partnerships and Working Group functions, and development of activities (see Supplement 1 for a topic guide used). Focus groups and interviews lasted for an average of 59 min. Data were collected until data saturation was reached [42], that is interviews and focus groups continued until basic themes were identified and confirmed by the research team, and additional data collected did not lead to any new emergent themes. As described by Bryman, [43] page 18, undertaking this reflective process, leads researchers to ‘combine sampling, data collection and data analysis, rather than treating them as separate stages in a linear process’.

Interviews and focus groups were audio-recorded and transcribed verbatim, and participants were de-identified. Data analysis was guided by the six phases of thematic analysis by Braun and Clarke [44], where researchers first read and became familiar with the data; initial codes generated; themes generated; themes reviewed; themes defined; and data written up. Data were analysed inductively, that is data were coded without trying to fit into a pre-existing coding framework [44]. Data were stored, coded, classified, and sorted using NVivo software (QSR International Pty Ltd, version 10, 2014). Data analysis was confirmed by a second member of the research team and, where required, incongruent results were managed by discussion among the whole research team to reach consensus. All methods were carried out in accordance with relevant guidelines and regulations, and study protocols approved by the Human Research Ethics Committee (Tasmania) Network (H0017793).

## Results

**Fig. 2:**
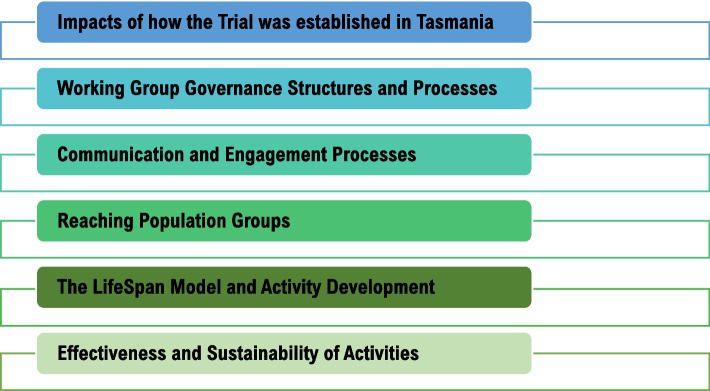
Six primary themes identified from focus group and interview data

Six key themes emerged from focus group and interview data (Fig. 2), presented alongside codes used to categorise data in Supplementary file 3.

Themes centred on the broader factors which stakeholders most reported impacted the implementation of activities under a systems approach in their communities. Themes are presented as per Fig. 2, with supporting quotes from the various participant groups, as followed. To ensure confidentiality, quotes are attributed at the level of participants’ broader group membership, being either Working Group member, Tasmanian Primary Health Network employee, or external stakeholder. As the evaluators of the program, no comparisons are made between sites, where the funding body explicitly requested that the trial site locations were to be treated as a single site.

### Impacts of how the Trial was established in Tasmania

Differing levels of understanding of the purpose of the Trial impacted how people established activities. As described by a Primary Health Network project staff member, decisions made by the TSPTAG, as higher-level project governance representatives, played a key role in how the Trial was established.

- *…they [the Advisory Group] had an integral and fundamental role to play in the design of the trial. You know, identifying our target populations, where the trial was geographically situated, and in approving some of the initiatives. – Primary Health Network 6, female*

In Break O’Day, a Working Group member described how having a host organisation already actively working in suicide prevention was perceived to have helped the establishment of the Working Group, this was commonly highlighted by participants across all the Working Group.

- *…having had the mental health action group on the ground and already involved in that space gave us a little bit of a head start, because there were already some layers that had been put down to help this work. – Working Group member 10, Break O’Day, male*

Another Working Group member from Break O’Day found voluntary and committed Working Groups members was crucial to the establishment of the Trial in the area, providing the motivation needed to overcome challenges, including bureaucratic/administrative burden.

- *The Working Group members’ voluntary approach, their passion for their community to enable this to be established, to bear with the process and the bureaucracy and continue, it’s a big commitment. – Working Group member 4, Break O’Day, female*

It was commonly acknowledged across participant groups that there was a need for people with appropriate local and specialised knowledge to sit at the table and develop feasible action plans, as described by this Primary Health Network employee.

- *“… for the Working Groups themselves to say, you know, have we got the right people around the table and if not, who else needs to be here? – Primary Health Network 6, female*

Across participant groups there were variations with how the Trial was understood, for example the following Working Group member understood the Trial as aiming to use different approaches to reach the specific population groups.

- *My understanding of the suicide prevention trial site is trialling different strategies and approaches to suicide prevention…a particular client demographic―men aged 45 plus and women 65 and older. – Working Group member 4, Break O’Day, female*

Whereas, this Primary Health Network employee understood the Trial would be an opportunity to explore a systems-based approach and how communities can utilise the selected LifeSpan framework to instigate positive change.

- *The trial is an opportunity to test the systems approach… looking at community capacity to influence systems across the board using LifeSpan. – Primary Health Network 6, female*

Although the Trial was largely understood to be looking at the capacity of communities and utilising novel approaches to reach priority populations, the following External Stakeholder, a service provider, had an outlying perspective that it was a contradiction that the model itself was administered through Tasmania’s Primary Health Network and therefore, still “dominated by traditional health paradigms”.

- *…how could we respond more effectively in terms of suicide prevention given those key target groups?… how could we develop the capacity of the community?…[the Trial was] a program that was about innovation and saying we need something different than traditional health paradigms… on the same hand, you’ve got an organisation [Primary Health Network], which is absolutely and utterly dominated by traditional health paradigms. – External Stakeholder 1, service provider, female*

### Working Group governance structures and processes

Across the Trial site there were standard governance structures guiding Working Group action, with several factors impacting how Working Groups planned and implemented suicide prevention activities in their local area. From establishment, the role of the Primary Health Network was seen across participant groups as essential to supporting the Working Groups, including the advisory (consultant) role, as described by these employees from the Primary Health Network.

- *There is a lot of bureaucratic hurdles that the working groups and the Coordinators had to jump over and the [Primary Health Network] Consultant could actually support the coordinator and the Host Organisation through that process. – Primary Health Network 1, female*
- *It was a lot of bureaucratic processes. You know, the action plans and reports and all of that and that we were able to provide that direct guidance around it. – Primary Health Network 4, female*

Tasmania’s Primary Health Network acted as a “conduit of advice”, an access point or “connector” for information needed by the Working Group for activity planning and implementation. This Primary Health Network employee provides the example of the crucial partnership established with the Coroner’s office.

- *The relationship that we built with the Coroner’s office, and, you know, for a Coroner’s office to offer an NGO access to some of the data they allowed us to access, you know, we had to prove to them that we could manage that data in a very sound and capable way…they may need organisations like a Primary Health Network or similar to enable that kind of conduit of advice. – Primary Health Network 5, male*

Working Group members across the Trial site discussed the administrative burden of implementing a systems-based approach at the community level. A more streamlined approach to bureaucratic processes was suggested as being needed, with the involvement of the Primary Health Network seen as an impediment to achieving this given the reporting requirements.

- *It’s been quite restrictive, and I’ve seen the admin work that had to be done. There’s a lot of bureaucracy…we can see things that need to be done and we want to do them and we’re not able to do them…it’s not a lack of us wanting to, it’s a lack of resources being given and powers-that-be holding back, for whatever reason. – Working Group member 8, Break O’Day, female*
- *In the initial stage things were quite highly directive, an unusual method…the level of hands-on by PHN [Primary Health Network] has been…not one I would endorse going forward… – Working Group member 12, Break O’Day, female*

The importance of streamlined processes to access resources to be able to initiate action was also mentioned from this External Stakeholder, a member of the Tasmanian Suicide Prevention Community Network.

- *Where you’re trying to get community-made action and suicide prevention, you need to make it as easy as possible…when they need resources, they need to be readily available alongside that. – External Stakeholder 10, Tasmanian Suicide Prevention Community Network member, male*

As explained by the following Primary Health Network employee, and supported across participant groups, many of these governance and administrative processes were driven by contractual obligations associated with participating in the Trial, thereby necessitating close involvement by the Primary Health Network to ensure due diligence was followed.

- *This is part of a national trial. And that brings with it certain obligations and ways of working and accountability…we can’t just let money go out the door without knowing what it’s for, the evidence for it, how it’s going to be expended? – Primary Health Network 6, female*

As described by the following Working Group members in the North-west, locally available social and physical capital influenced the makeup of the group and activity implementation.

- *…we’ve become more community-focused, so that the people who are now on the group were already working in the community, making those kinds of connections…it’s shifted from top-down to ground level, where the work is really being done. – Working Group member 19, North-west, female*.
- *We utilised our existing organisations such as Men’s Shed, Rotary events…Sporting clubs, annual dinners… we utilised those existing networks. – Working Group member 19, North-west, female*

Working Group members across Trial site locations described the role of the coordinator as central to Trial site operations, likening it to a relationship and management role, connecting the Trial with local communities.

- *It’s nearly like a manager… supporting … giving advice, information, empowering them [the Working Group]. – Working Group member 4, Break O’Day, female*.
- *We’ve been really lucky to have a coordinator that’s gone right through [the trial period] because I think one of the things that we often forget is rural and regional people, are about people. – Working Group member 24, North-west, female*

As described by the following Working Group member, lack of role clarity and decision-making structures manifested as inactivity, this was highlighted by all Working Groups.

- *All the Working Group meetings were just conversations, nothing really got done…We didn’t think we could make those decisions because it’d have to be a group decision, but then the group didn’t think that it was their responsibility to make those decisions. PHN [Primary Health Network] didn’t want to make those decisions because they’re a funder…it was conflicting. – Working Group member 2, Launceston, female*

When looking at Working Group structural factors that influenced progress, community members with lived experience were acknowledged as a powerful enabler to both the design and implementation of activities, bringing a different perspective and much-needed energy. This was highlighted across all participant groups, and described by the following Working Group member.

- *… they had the more ‘can-do’ approach that really changed the energy in the room…They came up with ideas… changed the way people thought about things… how do we make this work? How do we fix it?…How do we overcome it?” – Working Group member 2, Launceston, female*

One External Stakeholder explained how every member felt that they had a purpose to be involved in Working Group activities, whether that related to a job role or lived experience.

- *One of the things that I think was really missed in this trial as it was implemented here was actually picking up people who were genuine…Some of them had lived experience, some or everybody had some form of motivation to be involved… generally, they were picked up because they hold positions of some sort. – External Stakeholder 1, service provider, female*

### Communication and engagement processes

Throughout the Trial, communication and engagement processes centred around accounting for individual learning styles and community literacy levels. Several factors impacted these processes, which then impacted people’s understanding of the Trial and activities implemented, and ability to engage in these processes. A Working Group member in Break O’Day described effective and timely communication as being essential to community engagement and managing community expectations.

- *When it started off, the community engagement, there was a lot of work going on behind the scenes. I’ll be frank and say I don’t know that we have been on top of communicating our messages as we could to the community…when that happens after there’s been a launch in the expectations, people do need regular updates to see what’s happening. – Working Group member 12, Break O’Day, female*

Early communication of the Trial to the community was considered essential to enable timely implementation of activities. This is described by the following Working Group member in Break O’Day, who perceived that the involvement and role of Tasmanian Trial site as a part of the National Trial was not initially communicated in a clear or timely manner.

- *Why was the mainland announced and [Trial site location] wasn’t originally on there? And then we were later added… if we just knew straight up this is why we’re saying no to what we rather that you know where you stand, what you can work with and what’s reasonable. – Working Group member 5, Break O’Day, female*

As described by this Launceston Working Group member, the engagement processes within the Participatory Action Research evaluation approach helped Working Groups unpack the different components of the Trial, including the role of stakeholders and key relationships needed.

- *…all of those relationships, all of those times…the PAR process has been really good in unpacking some of those as well. – Working Group member 2, Launceston, female*

Different communication styles were described by the following Working Group member in Break O’Day as contributing to people interpreting things differently, a consideration when raising awareness of suicide and its prevention at the community-level, using a systems approach.

- *There were different communication styles and things like that, so that was also challenging at times. People interpreted things differently. – Working Group member 25, Break O’Day, female*

### Reaching population groups

Working Group members across the Trial site experienced challenges in engaging and providing activities under the LifeSpan model, for the specific target groups that were supposed to be the focus of Trial. In Break O’Day, the following Working Group members describes the barrier of larger geographical distances as impacting access to target group. An older population of 65 + may not use, or frequently access, email or social media. For this cohort it was found that traditional communication methods, for example direct communication via word of mouth, or radio, were most effective.

- *One of the barriers with such a widespread rural area … We have a community that…are an older population, so technology isn’t a way… [Host Organisation] uses the radio a lot… – Working Group member 25, Break O’Day, female*
- *…in our community, word of mouth seems to be the most effective. – Working Group member 4, Break O’Day, female*

Face-to-face activities were the most utilised activity format when engaging the male cohort. Taking the training to the priority groups workplace was described by this External Stakeholders, a member of the Tasmanian Suicide Prevention Community Network, as a method to help overcome participation barriers.

- *[Hold events] in an environment like a Men’s Shed…They come to the Men’s Shed because they’re isolated, they come there. They don’t want to go and see the professionals over there. – External Stakeholder 5, Tasmanian Suicide Prevention Community Network member, male*

In the North-west site, one Working Group member noted that engaging senior-level representatives from workplaces with large numbers of employees in the priority groups, for example to sit on Working Groups, could increase participation of employees from those organisations.

- *It would’ve been good to have had a HR [Human Resources] man from a large workforce…it’s all well and good to say, “We’ll go out and target male workplaces”. Good luck getting the employer to release these people from their paid jobs…backfill…pay other people to come along to training. Like, it sounds good in theory but in practice… – Working Group member 1, North-west, female*

The following Working Group member from the North-west recognised that to reach community members at risk under any of the LifeSpan strategies, conversations are needed between people not at the frontline of suicide prevention, but across sectors and communities, with community members well placed to be “gatekeepers”.

- *I had an email conversation with [allied health professional] in [coastal town] …she says, “I see suicidal people all the time because of their chronic pain and how it impacts on their life”. I’m like, “What do you do with them?” and she’s like, “I don’t know. I just, like, let them talk”. And I’m like, “Do you know there’s suicide [prevention/support] services who they can go and see for free?” And she’s like, “Oh, thank you” …so we’re not doing anything new but we’re just connecting. – Working Group member 1, North-west, female*

For the delivery of some activity types under LifeSpan, i.e., training or education, reaching those at risk meant being able to understand the needs of the target group an apply appropriate communication styles. This was described as finding the “right” voice, someone relatable to the cohort, to connect with community members, by this Working Group member.

- *… the recent activity with Doc Robinson, he’s not a professional speaker; he’s a dad, talking about his boy…they [the audience] trusted [him] despite the roughness of the delivery… on their own level… the vast majority of us going out and speaking is not going to reach the people that we talk about as being ‘unreachable’. – Working Group member 19, North-west, female*

### The LifeSpan model and activity development

Out of the nine LifeSpan strategies, Working Groups chose to build community capacity and awareness, i.e., through the “community engagement” strategy (see Fig. 1). Efforts mainly delivered through a third party to those in gatekeeper roles, rather than the at-risk population themselves, were preferred.

- *A lot of the activities have ended up being one step removed in most cases from the actual person at risk of suicide…it’s delivered to maybe a professional or someone else that might have contact with them. – Working Group member 14, Launceston, male*

Consistently highlighted across participant groups, and as described by the perspectives of a Launceston site Working Group members and a Primary Health Network employee, some strategies were considered overly ambitious at the community-level, due to lack of capacity, influence, or resources.

- *We’ve asked communities implementing the model to be able to have a significant influence in other areas, like emergency and follow-up care, treatment regimens and even improving safety and access to means…it would take a pretty strategic, well resourced, and capable leadership team to be able to do that…in a short time frame… – Primary Health Network 6, female*
- *…there’s a lot of things we’d love to do in [the] health sphere, and accident and emergency and you know, educate GPs and all that kind of stuff. Who in this room can do that? No one… – Working Group member 16, Launceston, female*

As described by the following Primary Health Network employee, when developing activities under the Lifespan model, a community co-design process is needed, working with communities to utilise and build existing resources and relationships, and recognising local needs may require only some strategies to be implemented.

- *Sometimes it’s not having all pieces of the pie covered…[It’s]codesigning with communities to do what communities do best in the context of what resources they have at hand. What relationships do they have? What are their priorities? Rather than sort of tasking them with delivering on the whole of that LifeSpan model. – Primary Health Network 6, female*

The LifeSpan systems model was perceived as a helpful starting point, providing valuable direction for suicide prevention activity planning. Overall, the model was seen as reflecting the broader areas of people’s lives which impact on suicide.

- *It enabled us to get some activities on the ground…it gave some direction. – Working Group member 1, North-west, female*.
- *It seems to encompass all parts of everybody’s lives… there’s room for help and improvement and support from every area of a person’s life, which is really what people need. – Working Group member 15, Launceston, female*

Barriers to implementing the full LifeSpan model centred on its perceived complexity, which was commonly discussed across participant groups. The following Working Group member described how the framework was perceived as a barrier to involvement of people with lived experience and people with low literacy in Working Groups.

- *I’m coming from a grassroots level of community development. It was a huge barrier to include people with lived experience, people that have never worked in an office in looking at that model…it is extraordinarily difficult for people with low literacy. It’s dis-empowering and what you’re losing there is some really rough diamonds that have valuable input and linkages to those that are vulnerable. – Working Group member 4, Break O’Day, female*

As highlighted by the following Working Group member and an External Stakeholder, despite being promoted as a whole of community response to suicide, the LifeSpan framework does not include a postvention strategy. Postvention was described as essential to reach those at risk after attempting suicide, as well as those bereaved by suicide, who are at higher risk themselves.

- *There was a gap in the model…there’s no postvention part of that model that deals with people who have attempted suicide and the people left behind. – Working Group member 3, Launceston, male*
- *It’s based around the whole community, response to suicide prevention, based around the Black Dog Institute model in nine sectors…you left out postvention… it’s actually a very significant part of suicide prevention. – External Stakeholder 9, Tasmanian Suicide Prevention Community Network member, male*

A member of the Tasmanian Suicide Prevention Trial Advisory Group noted that there was also lack of information on how to adapt the framework and accommodate for differences in gender, despite this being considered essential by Working Groups in understanding how to modify activities and overcome barriers to accessing at-risk groups, including men.

- *Gender was, for me, the missing ingredient…that’s also historically part of why we haven’t really been able to make big inroads on the numbers, because we’ve not had a gendered focus…programs and services that have been used, or funded in the past, such as Lifeline, tend to reach women better than they do men… we know 75% of suicide is in men. – External Stakeholder 3, Tasmanian Suicide Prevention Trial Advisory Group member, male*

A member of the national evaluation team recognised the role of the Primary Health Network as needing to take on aspects of community development when implementing the Trial.

- *I suppose in terms of PHN’s [Primary Health Network] knowledge and capacity around working this community and essentially doing community development work which was a bit sort of out of the realms of normal PHN business for a lot PHNs – External Stakeholder 14, National Evaluation team, female*

### Effectiveness and sustainability of activities

Participants explained how effectiveness was defined within the context of the Trial, and their communities, and how efforts could best be sustained in Tasmania post Trial. The following Primary Health Network employee and member of the Tasmanian Suicide Prevention Trial Advisory Group described success as being along a continuum, measuring outcomes other than suicide rates to reflect progress. The following highlights how relationships building, and increased awareness were considered as evidence of effectiveness of the Trial.

- *Every relationship that’s built, every effort that’s made to engage a service is progress. I think use “progress” rather than “success”…understanding the extent to which you were able to achieve what you set out to do…it’s teaching us something we can apply next time. – Primary Health Network 6, female*
- *The ultimate measure is the suicide rate [but]…there’s so much more you can measure. I mean just raising community awareness about how to talk safely and how to support people…getting a broader community to understand what the impacts of loneliness are…whole community awareness raising. – External Stakeholder 2, Tasmanian Suicide Prevention Trial Advisory Group member, female*

A Working Group member in Break O’Day perceived it as disconcerting that while measuring program effectiveness was a primary focus of the Trial, a reduction in suicide was not able to be measured within the life of the Trial in Tasmania. This was a commonly reported concern, particularly across Working Groups and external stakeholders.

- *I think that was a little bit disconcerting was that– when this was initially introduced, it was with the idea of being able to evaluate the reduction in suicide. – Working Group member 12, Break O’Day, female*

In Launceston, one Working Group member described how effectiveness was demonstrated through the partnerships established through the Trial, including inclusion of people with lived experience within the Working Group. Success was viewed as building the capacity of people representing the priority populations, also seen as a sustainability measure.

- *Our lived experience group…a partnership that’s happened because of the trial … the trial may, or may not be there after June but they, the learnings that they have and the confidence that they have, would still be there. – Working Group member 2, Launceston, female*
- Another Working Group member in Launceston described how the systems-approach called for focus on specific strategies, however this respondent felt that flexibility was central to the success of a systems approach. There was a perception that there was still flexibility in the model to focus on areas the local Council felt comfortable in, for example community capacity building, within suicide prevention efforts.
- *I didn’t like the sense that we were being pushed towards a particular part of the model…around the medical stuff within the hospital system… whereas if we were to work with our strengths as a council, we’d been working more towards the lived experience, towards more community capacity building. – Working Group member 3, Launceston, male*

Several Working Group members across the Trial site described how decisions were often based on getting started, even just one-off activities not considering sustainability. Partnerships, continued funding, and people’s capacity to take ownership were regarded as important elements to sustaining project outcomes.

- *A lot of those activities were just a one-off thing. But as we’ve gone on, we’ve built on some of those… It’s been partnerships, and we’re relying on each other to deliver both of our objectives. – Working Group member 2, Launceston, female*
- *Is it sustainable if someone takes ownership of it?… then try find an owner… – Working Group member 19, North-West, female*.
- *We’re up to our third event of working together and building those relationships with the Child and Family Centre, with us, with Mission, with the Church Group, with Rotary…that’s going to be somewhat sustainable going forward…that hinges on funding…it’s hard to do things when you’re penny-pinching. – Working Group member 1, North-West, female*

Building relationships with local services was considered a low-cost, high-impact practice for sustainability.

- *There was probably a conscious idea to work with and embed local services to … upskill, capacity build…and be involved in sustainability aspects, that’s what they’re trying to do. – Working Group member 12, Break O’Day, female*
- *They’re fairly simple strategies that don’t require a lot of money…build that level of sustainable relationships on the ground… – Primary Health Network 2, male*

Working Group members discussed how suicide prevention training and increased awareness led to changes to organisational culture and practices, including within population-group specific organisations, which could have a legacy post-Trial.

- *…to educate men in different workplaces… maybe that is an element that then is changing the work culture in those workplaces. – Working Group member 1, North-west, female*
- “*They’re really starting to embed in their practice as a [workplace], a lot of this work…this is going to continue beyond the pilot, regardless. – Working Group member 7, Break O’Day, female*

As discussed by a Working Group member in the North-west, there were advantages of drawing on the resources and expertise of local Councils, which was commonly recognised across the Working Groups.

- *Councils run events all the time, it would be very easy for them to put something in those events…They have a lot of levers to pull. Councils know their communities and the different pockets within their communities. – Working Group member 1, North-west, female*

It was evident from discussions with community members involved with the Trial that fatigue was a significant concern. Whether a trial or not, there was a need for program staff and communities involved to consider the lasting impacts of community fatigue when implementing a systems-based model.

- *These communities have built up a level of expectation…trials not continued or converted into more meaningful change, actually supported by dollars- that’s three potential [trial site] areas that are going to feel let down, particularly the people that have really been engaged in the process… that’s always an issue for trials, there’s always risks from a design component, like there’s uncertainty about what’s going to go ahead, you’re at risk of losing people because of that… – External Stakeholder 2, Tasmanian Suicide Prevention Trial Advisory Group member, female*

Post-Trial, there was a perception that government had responsibility for ensuring ongoing support for local communities, to continue suicide prevention efforts in their communities and build on Trial learnings.

- *The notion of a trial does suggest ideally that you’re going to do something afterward…we shouldn’t be having a trial unless there’s a recognition or an acknowledgment that we’re going to then implement what has worked. Otherwise, it just let’s communities down. It breaks trust. – External Stakeholder 3, Tasmanian Suicide Prevention Trial Advisory Group member, male*

## Discussion

As highlighted across participants, strict adherence to the Lifespan model, which was perceived as a top-down approach decided on by the government, was not possible within the proposed time or available resources in the communities. The establishment of the Trial in Tasmania and Working Group governance structures and processes impacted on activity development, adherence to the Lifespan model, and ability to reach target populations. This highlights a need for communities to implement longer term programs with sufficient planning and implementation time, and the need for comprehensive assessment of resources available to implement activities under a systems-model. Working Groups preferred to develop activities using a bottom-up approach, based on the availability and interests of people who could undertake initiatives, the best use of available time and resources, and being able to meet some of the perceived needs within communities. Working Groups preferred not to deliver activities only to defined population groups, believing the wider community should not be excluded. Activities therefore tended to focus on whole-of-community engagement, improved coordination of existing services, awareness raising, and community capacity building, to increase the confidence of communities to take action themselves. Continuous community engagement and community inclusion in key decision making was key to implementing suicide prevention efforts across the Trial site. The involvement of host organisations enhanced local community links with the Trial, as they were seen to be ideally placed to implement community level strategies and draw upon existing mental health or suicide prevention programs and networks.

It was recognised across participant groups that adaptable approaches to activity development and implementation were essential to ensuring that most strategies under the LifeSpan model were targeted. This was described as particularly important for those harder to implement strategies such as means restriction or influencing practices in primary health systems, where the influence, capacity and resources of Tasmania’s Primary Health Network were required to target these strategies. Continued Primary Health Network level involvement in the implementation of systems-based approaches will ensure a closer approximation of simultaneous implementation of all strategies in a model, as described as being critical for full impact [6, 7, 45].

Working Groups found delivery of activities to the priority population groups of men and older aged people challenging under a LifeSpan systems framework, with little influence within particular sectors to access and communicate with people at risk, i.e., aged care, and some strategies having little relevance to the population group, i.e., schools. Taking training to where men feel comfortable or in familiar settings (for example Men’s Sheds, and/or where they work), helped reach and secure participation of males aged 40–64. Such strategies increased access to awareness training and education to overcome stigma and assisted with logistical and financial barriers with attending training and activities outside of work hours. The role of people delivering training to men needed consideration, supporting previous research which has found that engagement and feelings of connection with a service/support provider plays a significant part in whether men access services or supports [46].

Across all activities delivered under the LifeSpan strategies, access to, or the means to use, digital communication, impacted on engagement of people aged 65 and over. These barriers have been previously explored, for example using digital communication and clinical treatment platforms and mobile apps [37, 47], however with additional insight of inaccessibility from being rurally located. The importance of understanding and accounting for literacy levels, including digital, health, mental health, and suicide literacy was pivotal for community engagement and activity implementation. Engaging aged care organisations in workplace training and resource distribution strategies used to reach older people at risk was integral. Peak bodies and service providers needed to be engaged from commencement for this to effectively occur. This further underscores the need of Primary Health Network-level intervention, to foster these necessary collaborations with senior level management in organisations that are crucial touchpoints for reaching people most at risk.

Working Group action and decision-making was strongly influenced by supporting governance structures and by capacity, knowledge, and experiences of community members and people with lived experience. In turn, these community members utilised the Trial to communicate program preferences based on their insights and knowledge about the needs of their local communities. While a focus on effectiveness and sustainability of activities was seen as important, there was limited potential to ensure these were included for every activity planned. Throughout the Trial, regularly reflecting on what worked well, and not so well, and adapting and improving processes as needed, including through the use of a PAR approach with evaluators, enhanced program planning within Working Groups and the Primary Health Network. Engaging community in program evaluation and program decision making is also essential to incorporate community perceptions and attitudes towards suicide and health, which are fundamental for program implementation and success. Insight from community and program stakeholders is key to influencing and shaping services and policy [31], ensuring program goals, and desired outcomes, best suit the communities that the program intends to service.

### Limitations

Several limitations of the current study should be considered when interpreting the findings. The number of participants varied across the three Trial site locations; however, data were combined to reflect the overall Tasmanian trial site experience. This may contribute to results being representative more of one site. Several processes varied across the Trial site regions, including how Working Groups were structured, their membership and levels of engagement of members, service organisation support, the processes used to recruit service organisations, and the level of community member representation and input into decisions. Caution should therefore be exercised when generalising findings across participant groups, or across the whole Tasmanian site. Due to a delay between commencement of the Trial and engagement of the evaluation team, and as funding for the evaluation ceased prior to the end of the Trial, stakeholders’ perceptions concerning the start and finish of the Trial could not be fully captured.

## Conclusion

Evaluations of suicide prevention programs in regional, rural, or remote areas are limited, particularly evaluations of programs using systems-based approaches, focusing on men and/or older aged individuals. This study contributes to the evidence showing systems-based approaches, the LifeSpan framework in particular, can be implemented in regional and rural areas, as long as several considerations are taken into account. There is a critical role for early community engagement, the inclusion of insight from people with lived experience, representatives from vulnerable population groups, peak bodies, and senior management of services on Working Groups. Implementation of systems-based approaches needs to consider potential barriers within a community, as identified by the community themselevs, which may impact being able to reach those at risk. There needs to be a focus on increasing the capacity of the whole of the surrounding community to recognise and support those at-risk. Sustainability of initiatives like the National Suicide Prevention Trial depends on continued community-ownership and involvement, timely access to resources and funding, and partnerships with local community, to understand local needs and support action at the community-level. Funding and implementation of longer-term programs and evaluations utilising systems-based models are needed in Australia, to measure the impacts of suicide prevention efforts on other priority populations, and on suicide related outcomes.

## Supplementary Information

**Additional file 1: Supplementary file 1. **Focus Group/Interview Topic Guide. Trial Site Working Group members, Coordinators, Host Organisation Managers and Primary Health Tasmania.

**Additional file 2: Table1. **Consolidated criteria for reporting qualitative studies (COREQ): 32-item checklist.

**Additional file 3: Supplementary Table 2. **Codebook from interview and focus group data showing themes and sub-themes.

## Data Availability

The datasets generated and/or analysed during the current study are not publicly available due to the confidential nature of the qualitative data and the ability to recognise individuals but are available from the corresponding author on reasonable request.

## Declarations

The Consolidated criteria for reporting qualitative research (COREQ), 32-item checklist for interviews and focus groups [48], is provided for this study in Supplementary file 2.

### Source

LifeSpan model(16) from Black Dog Institute.
