# Supplementary material for: Stakeholder insights into implementing a systems-based suicide prevention program in regional and rural tasmanian communities
Source: BMC Public Health. 2022 Dec 12;22:2323. doi: 10.1186/s12889-022-14721-5 (PMC9746171; doi:10.1186/s12889-022-14721-5)
Supplement: Supplementary file 1 — Additional file 1: Supplementary file 1. Focus Group/Interview Topic Guide. Trial Site Working Group members, Coordinators, Host Organisation Managers and Primary Health Tasmania. [file 12889_2022_14721_MOESM1_ESM.docx]

| **Supplementary Table 2 Codebook from interview and focus group data showing themes and sub-themes** |
| --- |
| **Theme 1. Understanding and Establishing the NSPT in Tasmania** |
| Awareness and understanding of the NSPT |
| Barriers to establishment |
| Enablers to establishment |
| How people got involved |
| Role of the Tasmanian Suicide Prevention Trial Advisory Group |
| **Theme 2. Working Group governance structures and processes** |
| Challenges within Working Groups |
| Establishing and maintaining Working Groups |
| Key Working Group members |
| Role of Black Dog Institute |
| Role of coordinator |
| Role of host organisation |
| Role of Primary Health Network |
| Supporting Working Group members with lived experience |
| Supporting Working Groups |
| The role of the chair |
| Working group funding and administration |
| Working Group governance and decision making |
| Working Group member expectations and contributions |
| Working Group structures and meetings |
| **Theme 3. Communication and engagement processes** |
| Communication and engagement with communities |
| Communication within Working Groups and Primary Health Network |
| Engaging local and contracted service providers |
| Engaging peak bodies |
| Engaging people with lived experience inc peak organisations |
| Suggestions for improving engagement processes |
| **Theme 4. Reaching population groups** |
| Barriers to targeting groups |
| Engaging target populations |
| Other potential priority groups |
| **Theme 5. The LifeSpan model and activity development** |
| Activity planning and LifeSpan strategies used |
| Activity planning for and involvement of priority populations |
| Barriers choosing and running activities |
| Factors influencing activity idea development |
| Necessary collaborations needed |
| Selection and adoption of LifeSpan |
| Suggestions for doing things differently |
| Understanding and perceived effectiveness of LifeSpan |
| **Theme 6. Effectiveness and sustainability of activities** |
| Measures of effectiveness |
| *Changes to service provision and use* |
| *Collaboration and capacity building* |
| *Community awareness, knowledge, capacity building* |
| *Community participation and engagement* |
| *Defining and measuring success and effectiveness* |
| *Difficulties with evaluating outcomes and measuring effectiveness* |
| Post-Trial and sustainability |
| *Disseminating learnings and managing community expectations* |
| *Support for trial sites post June 30 2021* |
| *Sustainability and activity planning* |
| *Thoughts on timing of trial and extension* |
| *Utilising findings* |
| Role of the evaluators |
| *Dual role and lack of role clarity* |
| *Using a Participatory Action Research approach* |
| *Role of local evaluators* |
| *Role of the national evaluator* |
|  |
|  |
|  |
|  |
|  |
|  |
|  |
|  |
|  |
|  |
|  |
|  |
|  |
|  |
|  |
|  |
|  |
|  |
|  |
|  |
|  |
|  |
|  |
|  |
|  |
|  |
|  |
|  |
|  |
|  |
|  |
|  |
|  |
|  |
