# Supplementary material for: Stakeholder insights into implementing a systems-based suicide prevention program in regional and rural tasmanian communities
Source: BMC Public Health. 2022 Dec 12;22:2323. doi: 10.1186/s12889-022-14721-5 (PMC9746171; doi:10.1186/s12889-022-14721-5)
Supplement: Supplementary file 2 — Additional file 2: Table1. Consolidated criteria for reporting qualitative studies (COREQ): 32-item checklist. [file 12889_2022_14721_MOESM2_ESM.docx]

| **Table 1: Consolidated criteria for reporting qualitative studies (COREQ): 32-item checklist** | | | |
| --- | --- | --- | --- |
| **No** | **Item** | **Guide questions/description** | **Author response** |
| **Domain 1: Research team and reflexivity** | | |  |
| **Personal Characteristics** | | |  |
| 1. | Interviewer/facilitator | Which author/s conducted the interview or focus group? | LG, TP, SA |
| 2. | Credentials | What were the researcher's credentials? *E.g. PhD, MD* | LG - MPH, BA/BCom, BPhil; TP - Bpsych (Hons); SA - MApp(Sci) |
| 3. | Occupation | What was their occupation at the time of the study? | Project Manager; PhD Candidate |
| 4. | Gender | Was the researcher male or female? | Female, Female, Male |
| 5. | Experience and training | What experience or training did the researcher have? | All researchers who conducted interviews and focus groups had extensive experience undertaking qualitative research and interviews and focus groups with vulnerable population groups |
| **Relationship with participants** | | |  |
| 6. | Relationship established | Was a relationship established prior to study commencement? | There was no relationship between the participants and researchers prior to the study/evaluation |
| 7. | Participant knowledge of the interviewer | What did the participants know about the researcher? e*.g. personal goals, reasons for doing the research* | Through reading the Participant Information Statement and as confirmed at the beginning of the interview or focus group, the participants were aware of the purpose of the study |
| 8. | Interviewer characteristics | What characteristics were reported about the interviewer/facilitator? e.g. *Bias, assumptions, reasons and interests in the research topic* | No conflicts of interest were disclosed by any of the authors |
| **Domain 2: study design** | | |  |
| **Theoretical framework** | | |  |
| 9. | Methodological orientation and Theory | What methodological orientation was stated to underpin the study? *e.g. grounded theory, discourse analysis, ethnography, phenomenology, content analysis* | The study used grounded theory. The evaluation used a Participatory Action Research approach, please see Methods for further information. |
| **Participant selection** | | |  |
| 10. | Sampling | How were participants selected? *e.g. purposive, convenience, consecutive, snowball* | Purposive and snowball, please see Methods for further information |
| 11. | Method of approach | How were participants approached? e*.g. face-to-face, telephone, mail, email* | Participants were approached through email |
| 12. | Sample size | How many participants were in the study? | 46 |
| 13. | Non-participation | How many people refused to participate or dropped out? Reasons? | Nobdy refused to participate or dropped out |
| **Setting** | | |  |
| 14. | Setting of data collection | Where was the data collected? e*.g. home, clinic, workplace* | Participants were located in a range of settings across the three trial site regions including workplaces, public spaces |
| 15. | Presence of non-participants | Was anyone else present besides the participants and researchers? | There was no one else present at the interviews and focus groups besides the participants and researchers |
| 16. | Description of sample | What are the important characteristics of the sample? *e.g. demographic data, date* | Participants comprised 46 people involved with the trial in Tasmania, including Working Group members (n=25) across all three site locations (North-West Coast, Launceston and Break O’Day (Figure 3), project staff at Tasmania’s Primary Health Network (n=7), and several external stakeholders, including the national evaluation team, peak suicide prevention body representatives, Advisory Group members, and members of the Tasmanian Suicide Prevention Community Network (n=14). Participant ages ranged from 28 to 75 years (median=53 years, SD=11.7). Just over half female (51.1%) and half (53.3%) reported a lived experience of suicide. |
| **Data collection** | | |  |
| 17. | Interview guide | Were questions, prompts, guides provided by the authors? Was it pilot tested? | A topic guide was utilised by researchers and the questions were pilot tested |
| 18. | Repeat interviews | Were repeat interviews carried out? If yes, how many? | No repeat interviews were carried out |
| 19. | Audio/visual recording | Did the research use audio or visual recording to collect the data? | Interviews and focus groups were audio recorded |
| 20. | Field notes | Were field notes made during and/or after the interview or focus group? | Field notes were taken by the research team at interviews and focus groups |
| 21. | Duration | What was the duration of the interviews or focus group? | On average, interviews and focus groups were 59 minutes |
| 22. | Data saturation | Was data saturation discussed? | Data saturation was discussed by the research team in regular team meetings and regularly by those collecting and analysing data. |
| 23. | Transcripts returned | Were transcripts returned to participants for comment and/or correction? | Due to the nature of focus group data and confidentitality issues with small groups, focus group data was made available only on request. No focus group or interview participants requested data. |
| **Domain 3: analysis and findings**z | | |  |
| **Data analysis** | | |  |
| 24. | Number of data coders | How many data coders coded the data? | Data were coded by two researchers and confirmed by a third. |
| 25. | Description of the coding tree | Did authors provide a description of the coding tree? | Authors provide a table/coding tree in additonal Supplement |
| 26. | Derivation of themes | Were themes identified in advance or derived from the data? | Themes were derived from the data |
| 27. | Software | What software, if applicable, was used to manage the data? | Nvivo spftware was used to analyse data |
| 28. | Participant checking | Did participants provide feedback on the findings? | Participants provided feedback on the findings at presentations made by the researchers including the Tasmanian Suicide Prevention Community Network and the Tasmanian Suicide Prevention Committee meetings. |
| **Reporting** | | |  |
| 29. | Quotations presented | Were participant quotations presented to illustrate the themes / findings? Was each quotation identified? e*.g. participant number* | Quotations represent themes and each quote was identified with participant number and group representation |
| 30. | Data and findings consistent | Was there consistency between the data presented and the findings? | The findings were consistent with the views presented across the participant group,and were the strongest in terms of how long participants spoke of these |
| 31. | Clarity of major themes | Were major themes clearly presented in the findings? | Six themes were identified impacts of how the Trial was established in Tasmania; Working Group governance structures and processes; communication and engagement processes; reaching priority population groups; the LifeSpan model and activity development; and the effectiveness, reach and sustainability of activities. |
| 32. | Clarity of minor themes | Is there a description of diverse cases or discussion of minor themes? | Diverse cases and minor themes are discussed as they are identified throughout the manuscript |
