# Supplementary material for: Stakeholder insights into implementing a systems-based suicide prevention program in regional and rural tasmanian communities
Source: BMC Public Health. 2022 Dec 12;22:2323. doi: 10.1186/s12889-022-14721-5 (PMC9746171; doi:10.1186/s12889-022-14721-5)
Supplement: Supplementary file 3 — Additional file 3: Supplementary Table 2. Codebook from interview and focus group data showing themes and sub-themes. [file 12889_2022_14721_MOESM3_ESM.docx]

**Supplementary file 1**

**Process Evaluation of the National Suicide Prevention Trial Tasmanian Sites**

**Focus Group/Interview Topic Guide**

**Trial Site Working Group members, Coordinators, Host Organisation Managers and Primary Health Tasmania**

Context: The Centre for Rural Health at the University of Tasmania is undertaking an evaluation of processes associated with the design, establishment and operation of governance structures within each of the three National Suicide Prevention Trial (NSPT) sites in Tasmania.

**ICEBREAKER**

1. What is your understanding of the NSPT program and can you tell me how you first got involved with the NSPT Program? (Interview only question)

**DESIGN**

1. What is your understanding on how the Working Groups came about/were established? What are your thoughts on this approach?
2. What are your thoughts about the adoption of the LifeSpan model as the preferred approach in the design and implementation of suicide prevention activities in your community?
3. How have local service providers, stakeholder groups, the broader community (including those members with lived experience of suicide) and other partnerships/collaborations been engaged in the design of program governance structures and processes? Are there any other stakeholders that should have been involved, and why?
4. What were some of the barriers and enablers experienced with targeting activities to the specified population groups? What strategies were employed to manage these?

**ESTABLISHMENT**

1. To what extent has the NSPT program established itself within the local suicide prevention landscape?
2. What do you consider to be some of the key enablers and barriers to how it has been established?

**OPERATIONAL**

1. What do you consider as some of the strengths and/or weaknesses of the internal structure and processes influencing the operation of the NSPT at the local level? (for example (prompts if needed) communication processes, decision making autonomy).
2. How have you found efforts by the funding body (PHT) to communicate with, guide, advise, and support working groups?
3. How have you found efforts by the trial site coordinator position to communicate with, guide, advise, and support working groups?
4. Could anything be done to better support the operations of the working group? If so, what?
5. What are your thoughts about the approach taken by the local evaluation team? In your opinion, what worked well/not so well?

**OUTCOME AND SUSTAINABILITY**

1. In your opinion, has the NSPT been effective in your community? Why/why not, and how? (for example (prompts if needed) local partnerships/collaborations etc).
2. Has sustainability of activities been considered in action plan development and activity planning? If yes, how?
3. Are there any further comments you would like to make?
